# Current Technological Improvements in Enzymes toward Their Biotechnological Applications

**DOI:** 10.3389/fmicb.2016.00965

**Published:** 2016-06-16

**Authors:** Mehak Baweja, Lata Nain, Yutaka Kawarabayasi, Pratyoosh Shukla

**Affiliations:** ^1^Enzyme Technology and Protein Bioinformatics Laboratory, Department of Microbiology, Maharshi Dayanand University, RohtakIndia; ^2^Division of Microbiology, Indian Agricultural Research Institute, New DelhiIndia; ^3^National Institute of Advanced Industrial Science and Technology, TsukubaJapan

**Keywords:** metagenomics, psychrophiles, protease, site-directed mutagenesis, DNA shuffling, enzymes

## Abstract

Enzymes from extremophiles are creating interest among researchers due to their unique properties and the enormous power of catalysis at extreme conditions. Since community demands are getting more intensified, therefore, researchers are applying various approaches *viz.* metagenomics to increase the database of extremophilic species. Furthermore, the innovations are being made in the naturally occurring enzymes utilizing various tools of recombinant DNA technology and protein engineering, which allows redesigning of the enzymes for its better fitment into the process. In this review, we discuss the biochemical constraints of psychrophiles during survival at the lower temperature. We summarize the current knowledge about the sources of such enzymes and their *in vitro* modification through mutagenesis to explore their biotechnological potential. Finally, we recap the microbial cell surface display to enhance the efficiency of the process in cost effective way.

## Introduction

**FIGURE 1:**
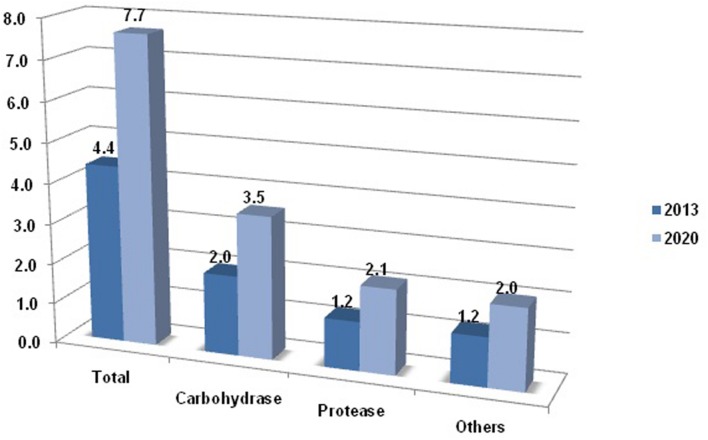
**The market share of microbial enzymes**.

As proven through research, living creatures are omnipresent, leaving no space vacant ranging from hydrothermal vents to glaciers. Some species have adapted themselves to an extreme environment, by acquiring range of adaptations for survival, at each level of cell function and structure (Feller, 2013). This unique attribute has created keen interest among the researchers to resolve the mystery of such living organisms. With the course of study, it has been found that microorganisms possess some membrane specialized structures and the proteins that enable them to proliferate in the extreme environment. Such microorganisms, possessing unusual cell makeup, are now proving as assets to our industries, enabling industrial processes to work beyond the normal range. For example, the cold-active enzymes provide the liberty to conduct the chemical reaction at fairly good reaction rate at low temperatures. The reason behind their enhanced catalysis at low temperature is because of the optimization of their electrostatics at or nearby active site. From few past years, industries are also showing interest in extremozymes. The increasing usage of cold-active proteases, particularly in the detergent industry, is because of their catalysis at low temperature, resulting in additional benefits such as cost reduction due to minimization of energy and retention of fabric quality, which was missing during the use of phosphate. The thermolability of psychrophilic enzymes strengthens the process by virtue of curtailment of unwanted side reactions. Owing to the fact that at such low temperature other enzymatic reaction ceases, further, employment of substrates and other components targets only the goal reaction. These atypical properties place the cold-active enzymes on top in industrial demand list, as the market competes for cost effectiveness and quality product. According to recent reports, the carbohydrases account for the largest market share followed by proteases among all enzymes, as shown in **Figure 1**.

The escalating demand for biocatalyst has put up challenges to modern biotechnology. These challenges can be met in either of two ways (i) improving the catalytic activity of the already existing enzymes, (ii) finding the novel enzymes. In this review, we describe these two approaches to obtain useful and applicable enzymes.

## Improvement of the Existing Enzymes or Proteins

For improving the existing enzymes, there are two approaches that allow redesigning of the enzyme, which may either enhance the activity or limit the inhibitory challenges of the enzyme (1) Rational redesign- as the name suggests it redesigns the existing biocatalyst using site-directed mutagenesis, thus, requiring full knowledge of 3-D protein structures and the mechanism of enzymatic reaction (2) Random mutagenesis method such as directed evolution, it mimics the natural process of variant generation and includes all molecular techniques for variant generation such as repeated oligonucleotide directed mutagenesis, error prone PCR, and chemical agents.

### Overview

Keeping in view the sustainable development, end users’ interest has shifted from chemical products to enzymatic products. The increasing interest in using enzymes for industrial processes has spurred the search for biocatalysts with new or improved properties by incorprating some imminent technologies of gene editing and novel bacterial platform for therapeutic enzymes (Gupta and Shukla, 2015, unpublished). Unfortunately, naturally available enzymes are usually not optimally suited for industrial applications because these enzymes are unable to withstand the extreme industrial conditions. Traditionally, the enzymes were optimized using statistical approaches like response surface methodology and Taguchi (Shukla and Gupta, 2007; Shukla et al., 2007). With progress in technology, it has become possible to edit peculiar residues in the natural enzyme to obtain a better enzyme with improved functional features. This can be achieved by high-throughput technologies or exploiting extraordinary sources *viz.* extreme environment so that their enzyme could sustain and perform better in extreme conditions.

Protein engineering is proving to be one of the successful technological approaches in biotechnology, being capable and able to generate a valuable intellectual property. The motive behind these methodologies is to surmount the snag of natural enzymes and fine tune to system-specific biocatalysts. In protein engineering, mutation is the key to explore protein function by mutating either at peculiar sites to achieve some new functions or design completely novel product that might give spectacular results for the better fit into the process. Protein engineering also deduces structure-function relationship. There are two main methods of protein engineering, site-directed mutagenesis, and random mutagenesis.

### Improvement by the Site-Directed Mutagenesis

Advancement in the area of enzyme modification has enabled tuning of a biocatalyst to meet an industrial objective. Site-directed mutagenesis is one such tool to build novel proteins that serve as efficient catalysts. It involves editing of an amino acid at a particular specific site and evaluating the effect of mutated protein, thus, the method is a choice for those proteins whose structure and mechanisms of action is already known. An advantage is that it takes less time in evaluation since the number of variants produced is less. It aids in evaluating structural and functional aspects of particular amino acid residues in a protein. The major application of site-directed mutagenesis is to introduce novel properties like enhanced specificity, stability, activity, solubility, expression etc. to the biocatalyst. A study was conducted in an attempt to improve the properties of α-galactosidase, a novel gene from deep sea bacteria *Bacillus megaterium*, was cloned and mutated. The study not only helped in improving properties of the enzyme but also gave tremendous structural-functional information that also revealed a mechanism of increase of activity at the molecular level. They found a protein that contains a tunnel structure, and the NAD (cofactor) makes a way to the active center via this tunnel protein (Xu et al., 2014). Recently, the approach has been applied to plant crops to introduce mutation using site-directed nucleases. It has an advantage of faster and controlled genome editing using directed mutagenesis, gene replacement, and transgene insertion (Collonnier et al., 2015). Site-directed mutagenesis also extends its application to the immobilized enzymes. Earlier immobilization was used to preserve enzyme function by using support attached to the enzyme in a random manner. Since the orientation of enzyme plays a significant role in the catalysis, therefore, researchers realized to immobilize the enzyme in a specific orientation with some specific residues. There have been few reports where enzyme cannot be immobilized due to their ionic hindrances, site-directed mutagenesis has enabled such enzyme to get immobilized. An example, where penicillin G acylase from *Escherichia coli* made to be adsorbed on DEAE or polyethyleneimine-coated supports by introducing eight Glu residues by site-directed mutagenesis. It was often seen that enzymes lose certain properties like thermostability etc. after immobilization. Site-directed mutagenesis has successfully culminated such limitation by incorporating specific residues at specific sites. The thermostability of the immobilized protease was improved by introducing Cys residues on surface of a cysteine-free mutant of a thermolysin-like protease from *B. stearothermophilus* and thus facilitated the site-directed immobilization of protease via single thiol group onto thiol-Sepharose (Eijsink et al., 1995).

An another attempt was made to improve stability and catalysis of thermolysin by substituting the three amino acids which lead 5 to 10-fold improvement in *N*-[3-(2-furyl)acryloyl]- glycyl-L-leucine amide (FAGLA)-hydrolyzing activity and *N*-carbobenzoxy-L-aspartyl-L-phenylalanine methyl ester (ZDFM) -hydrolyzing activity as compared with wild enzyme (Yasukawa and Inouye, 2007). Likewise, a single amino acid change in the amylase introduced resistance toward chemical oxidation. This mutant amylase was found to be highly compatible with the detergents (Chi et al., 2010). In another attempt to enhance uridyltransferase activity, mutations were introduced into the amino acid residues located within the predicted reaction center. Among twelve, six mutants successfully were found to have increased GlcNAc-1-P UTase activity (Zhang et al., 2007). In a first-ever study, to understand the molecular mechanism of the thermophilic archeal protein ST0452, isolated from *Sulfolobus tokodaii*, possessing glucosamine-1-phosphate (GlcN-1-P) AcTase activity and galactosamine-1-phosphate (GalN-1-P) AcTase activity, which is not detected in other proteins. Several types mutants were built, after analyzing their 3-D structures. After analyses, the researchers identified certain amino residues important for the both activities *viz.* His. 308 is essential for both GalN-1-P and GlcN-1-P AcTase activities, whereas Tyr311 and Asn331 are important only for the GalN-1-P AcTase activity (Zhang et al., 2015).

### Improvement by the Random Mutagenesis

Random mutagenesis follows the unbiased approach for variant generation mimicking the natural process. The nature takes years to evolve, by mutation or recombination and selects on the basis of survival of the fittest one. This technique provides an opportunity to give a mutated product in weeks by generating a library of mutants and selection of members on the basis of desired respective property. It also overcomes the blockade for the proteins whose structure or catalytic mechanism is not fully known. This technique demands an efficient, high-throughput screening system since a number of variants produced are often high in number. The technique introduces random mutations in a gene, with the main objective to characterize the open reading frames, generating a diversity of variants that are subjected to screening for the respective properties (Ramli et al., 2011). This indiscriminate mutagenesis in genes is based on two methods, i.e., *in vitro* directed evolution and gene recombination. The *in vitro* directed evolution/ random mutagenesis is performed by various techniques, like chemical mutagenesis, site-saturating mutagenesis, error-prone PCR, using mutator strains whereas, the techniques based on gene recombination are DNA shuffling, staggered extension process (StEP), random chimeragenesis on transient templates (RACHITT), iterative truncation for the creation of hybrid enzymes (ITCHY), recombined extension on truncated templates (RETT) (Sen et al., 2007).

**FIGURE 2:**
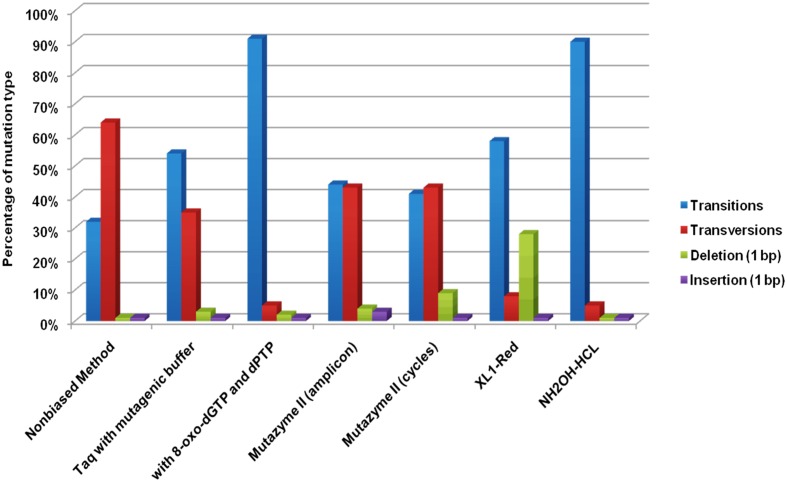
**Mutation types induced by the evaluated random mutagenesis methods (Rasila et al., 2009)**.

Although, there is no conclusion which method is best suited for a particular mutation. However, most commonly used is, error-prone PCR, where success is based on the error rate of Taq polymerase or alteration in the concentration of dNTP’s, Mg ions, an addition of nucleotide base analogs, using mutazyme polymerase during PCR (Kuddus and Ramteke, 2011). For example, the acid tolerance of *Lactobacillus pentosus* ATCC 8041 was significantly improved by using amplification of its genomic DNA using random primers and Taq DNA polymerase in a single cycle of mutation. The mutant yielded 95% of lactic acid in a medium of pH 3.8 whereas the wild strain was unable to grow at such low pH (Lidan et al., 2013). In another study, the thermostability of maltogenic amylase MAUS149 was improved using error-prone PCR (Mabrouk et al., 2013). There are few reports on the improvement of proteins by utilizing both random and site-directed mutagenesis in a single process. Like, a study in which the influenza vaccine strains were improved from the classical method, using error-prone PCR, site-directed mutagenesis and reverse genetics. The strains provided complete protection against influenza A(H1N1)pdm09 virus in mouse (Ye et al., 2015). In a study, the combination of error-prone PCR and DNA shuffling was used to produce variants of Cyclodextrin glucanotransferase enzyme to obtain higher product specificity for CD_8_ and a broad pH activity range. Compared to the wild-type enzyme which is inactive below pH 6.0, a variant retained 70% of its CD_8_-synthesizing activity at pH 4.0 (Melzer et al., 2015). A study was conducted in which, the error rate was enhanced during PCR up to 1.8 × 10^-3^ errors/bp using heavy water as a solvent instead of normal water using rhodopsin cDNA of the Ayu fish as a template (Minamoto et al., 2012). The error rate was improved in a study conducted by Rasila et al., 2009 to decipher the highest mutation rates among the various directed evolution method. It was deduced that highest mutation rates were observed among error-prone PCR methods. A comparison of various directed evolution methods has been depicted in **Figure 2**.

Sometimes, it has been observed that mutation causes improvement in one property while simultaneously compromise in other property. A breakthrough of this limitation is to “breed” protein with the suitable individual property and then screen “progeny” for the desired set of properties (Jon et al., 1999). The engineered protein formed after mutation depends on the quality of the library. One variant (3-2G7) of subtilisin S41 (psychrophilic protease) was created by random mutagenesis, saturation mutagenesis, and *in vitro* recombination/DNA shuffling, a remarkable improvement in temperature range was observed without compromise in its catalysis at low temperatures, and developed threefold higher catalytic efficiency (Lillford and Holt, 2002). Further generations of this enzyme exhibited even greater activity and stability (Struvay and Feller, 2012). The DNA shuffling is also proving as quite efficient in a diversity of applications. For example, the antifungal activity of *Lactobacillus plantarum* IMAU10014 strain was improved by three rounds of DNA shuffling. The mutant was effective up to 200% compared to wild strain with broad anti fungal spectra and bears good candidature for biopreservation (Wang et al., 2013). In another study, The thermostability of feruloyl esterase (FAE) was improved up to 22-fold after DNA shuffling with four homologous FAEs (Li J.J. et al., 2015). Further, salt tolerance in yeast was improved using two homologous Na^+^/H^+^antiporters from halophytes *Salicornia europaea* (SeNHX1) and *Suaeda salsa* (SsNHX1). The mutant exhibited up to 46% salt tolerance compared to parent strain (Wu et al., 2015).

### Example of Improvement of the Protease

The use enzyme has grown so fast in the industries, thus, there is continuous need to evolve the enzymes. Proteases hold largest market share accounting 52% of total enzymes. Protease applications are also too diverse ranging from therapeutics to detergent industry to dehairing, bioremediation etc. There are certain examples that show the versatility of protease different areas. A cardiovascular drug, nanokitanase is a bacterial serine protease that showed an improved catalytic efficiency and stability after mutation by site-directed mutagenesis. The double mutant showed the best results followed by single mutants and wild type. The study contributed to broaden their utilities in medical and commercial applications (Weng et al., 2015). Random- and site-directed mutagenesis on Harobin, a serine protease, with fibrinolytic activity and anti-thrombosis effect, enhanced the fibrinolytic activity. The mutant possessed much higher fibrinolytic activity and anti-thrombosis effect than wild-type enzyme with no detectable side effects (Li Z. et al., 2015). An another serine protease with its application in harsh washing, improved with the aid of site-directed mutagenesis. The mutation was made in the N-terminus region evaluated by sequence alignment and homology modeling, enhanced the catalytic efficiency, thermal stability and substrate affinity (Jaouadi et al., 2010). The mutagenesis has extended the application of proteases to an another level like dehairing capacity of the serine protease has been increased compared to wild type using a physical method of random mutagenesis, i.e., UV, *N*-methyl-N¢-nitro-*N*-nitrosdguanidine and Co(60) gamma-rays (Wang et al., 2007). A random substitution in the catalytic triad of subtilisin protease increased its activity at low temperature, turning mesophilic protein to psychrophilic protease (Kano et al., 1997).

### Improvement in Cold-Active Enzymes

The improvement among such enzymes is achieved by their effectiveness at low temperature and offers various advantages to the enzymatic process. The reactions catalyzed by the cold active enzyme are proved to be cost effective since heating during the process is curtailed, higher yield is obtained from the reactions involving thermosensitive compounds etc. In an study, the cold active β-glucosidase isolated from *P. lutea* BG8 successfully converted cellobiose to ethanol with 91.42% (0.49 g ethanol per g cellobiose) fermentation efficiency at 4°C (Tiwari et al., 2014).

The improvement in naturally occurring cold active enzymes may add additional benefits to the enzyme by employing site-directed mutagenesis and directed evolution methods. Like in a study, there was a simultaneous increase in both activity and stability of a psychrophilic lipase isolated from *C. antarctica* using directed evolution (Zhang et al., 2003). Another study was intended to improve the cold adaptation of alkaline protease, where error prone PCR was used. The activity of mutant product was successfully enhanced. The site-directed mutagenesis was also done to decipher the key amino acid involved in enhancing the cold adaptation (Liu et al., 2014b). Similarly, the catalytic efficiency of cold active purine nucleoside phosphorylase was increased by 1000-fold after site-directed mutagenesis (Xie et al., 2012).

In fact, studies are being done to improve the thermostability of psychrophilic enzymes with an objective to enhance the stability range. Kulakova et al. (2003) using rational mutagenesis deciphered the improvement in the cold-active serine alkaline protease from *Shewanella* sp. Similarly, in another study the thermostability of alanine racemase from *B. psychrosaccharolyticus* was improved by replacing Glu150 and Arg15 with Val and Ala at nearby active site (Yokoigawa et al., 2003). In an another study, the thermostability of cold active lipase from *Pseudomonas aeruginosa* was improved upto sevenfold by mutating in the region of high flexibility using rational design approach (Cesarini et al., 2012).

## Mining of the Novel Genes Encoding the Target Enzyme or Protein

### Overview

For recovering the novel enzymes, metagenomics, a culture-independent approach, has become a blessing to modern biotechnology. Metagenomics consist of isolation of genomic DNA directly from an environmental sample, that is analyzed by high-throughput sequencing *viz.* shotgun sequencing, 454 pyrosequencing, that minimizes the loss of important entities during the culturing. Metagenomics approaches are used to analyze the structure and functional potential of the microbial community. The structural analyses of gene/species richness, distribution etc., is often done by sequence-based screening whereas the functional potential of environmental microbial communities is evaluated via functional based screening. Metagenomics opened the gateway for the extremophiles that were almost impossible to cultivate and also bestow with novel enzymes of industrial value.

### Importance of the Environment

There is a continuous phase of adaptation in a life of extremophiles due to lack of hospitality in the environment. As extremophiles are surrounded by unusual environmental condition, the components that mainly suffer are enzymes and the lipid membrane. Because, these enzymes and membrane require certain flexibility to function, therefore the enzyme activity and transportation through the membrane is hampered in extremophiles. Thus, in order to sustain, such strains tend to adapt unusual makeup that makes them unique from the population of a hospitable environment. These unusual cells or genetic makeup is proving as an asset to our industries and a continuous effort is being laid to exploit maximum from them. Since metagenomics principle is based directly on an environmental sample, it is proving as one of the powerful tools to conquer these unusual proteins. Inference of comparative structural study of organisms living in different conditions concludes that their features totally contrast from each other. For example, a comparison of the temperature related constraints and their adaptive features are listed in **Table 1**.

**Table 1:** An indicative list of various constraints and adaptations among psychrophiles.

S. No.	Type of Constraints	Low temperature	Psychrophilic adaptations	High temperature	Thermophilic adaptations
1	Membrane fluidity	Decreased	Poly Unsaturated fatty acids and shorter acyl chains	Increased	Saturated and branched fatty acids and longer acyl chains
2	Transport of nutrients and waste	Altered	Specialized lipid components and low hydrophobic membrane proteins (Kahlke and Thorvaldsen, 2012)	Altered	High hydrophobicity in membrane proteins.
3	Activity of transcriptional and translational enzymes	Reduced	Cold-shock proteins (Csps), increased level of nucleic acid binding proteins and chaperons Dna K and GroEL (Amico et al., 2006)	Reduced	Heat shock proteins (Hsps), chaproninsGro EL, Gro ES, TCP-1, TriC, CCT
4	Protein folding	Misfolded structures	Cold shock proteins (Csps)	Misfolding	Heat shock proteins (Hsps)
5	Solubility of gases	Reduced and formation of reactive oxygen	Enhanced antioxidant activity by catalase and superoxide dismutase		
6	Intracellular ice formations	Very high	Anti-freeze proteins (Amico et al., 2006)	–	–

**FIGURE 3:**
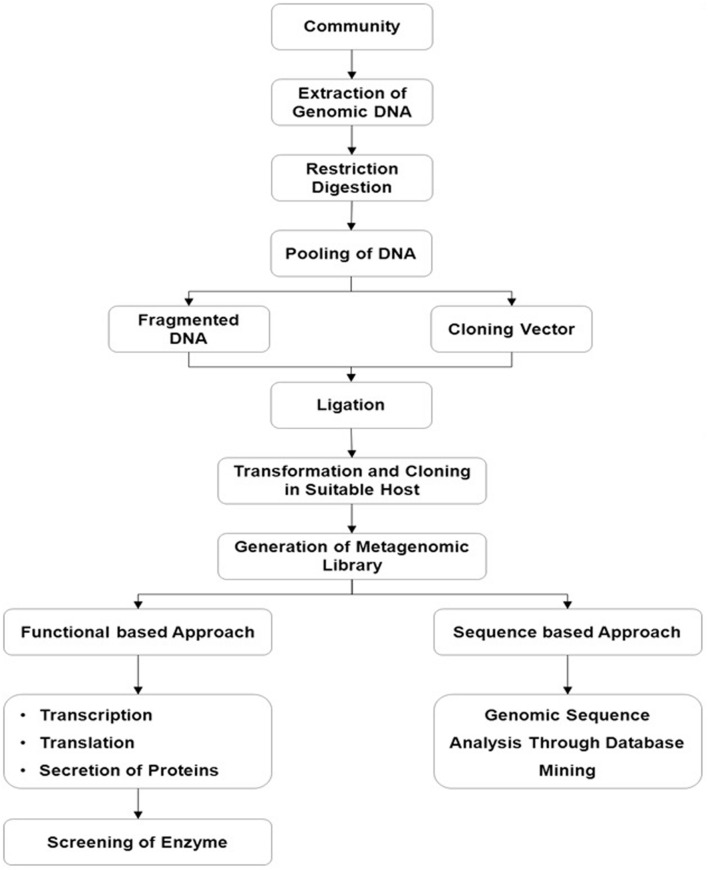
**Metagenomic based efficient enzyme screening in conjunction with functional based and sequence based approaches**.

Depending on the aim of the study, the selection process is usually based on (i) sequence driven screening, (ii) function-driven screening, and (iii) randomly mass sequencing, so-called metagenomics (**Figure 3**).

### Sequence-Dependent Identification of the Novel Gene

Sequence-based approach confides on sequence analysis for the conjuncture of function. The importance of the sequence based method lies in analyzing evolutionary relationships, linking phylogeny and function, identifying the unknown species, and detection of an abundance of genes. The oligonucleotides or probes are designed for the sequences encoding conserved domains of protein on the basis of consensus sequences (Sabree et al., 2009). The target ORFs are then identified by PCR or hybridization. The 16S ribosomal RNA gene (16S rDNA) is the most widespread phylogenetic marker used for identifying genome fragments derived from specific groups of microorganisms. However, it reflects only the phylogenetic classification of respective bacteria and not necessarily the metabolic function of the organism. For evaluating the specific gene from metagenome, the conserved gene sequence called ‘anchors’ are used and clones are identified by either hybridization or PCR.

For the identification of ORF similarity search algorithms (e.g., BLAST, COG, KEGG) provide sufficient information. Software tools are also now available to query large sequence datasets such as genomes and metagenomes for the presence of gene clusters associated with biomolecules of interest. One such tool, antiSMASH (the antibiotic and secondary metabolite analysis shell), that quickly identifies and annotates secondary metabolite gene clusters from genomic sequence data (Jackson et al., 2015). Further, hybridization also allows the handling and screening of a huge number of clones. A novel polyketide synthase gene from soil metagenomic clones was successfully detected by high-throughput DNA hybridization using functional genes as radio-labeled probes (Jacquiod et al., 2014). Large-scale sequencing projects such as the metagenome of the Sargasso Sea resulted in the identification of numerous novel genes by sequence-based metagenome analyses (Venter et al., 2004).

In a study, a novel bacterial laccase gene designated as lac21 was screened from a marine microbial metagenomic library of the South China Sea based on sequence screening strategy. The laccase gene has tremendous potential in decolorization of azo dyes in the absence of redox mediators with a comparatively lower level of supplementation (15 U/L) at 20°C (Fang et al., 2012). In a similar study, from hot spring of Manikaran, a pectinase gene was isolated. The enzyme was found to be thermostable with its optimum temperature of 70°C and stable up to 60°C for 5 h (Singh and Shukla, 2012). Similarly, xylose isomerase gene was found from soil metagenome (Nurdiani et al., 2015). Thus, metagenomics aids in novel discovering extremozymes that might not be explored by conventional cultural methods.

### Function-Based Identification of the Novel Gene

Functional screening is an alternative approach to sequence-based screening that does not require prior knowledge of sequence. The concept of function-based screening relies on expression of a metagenomic gene of interest in a surrogate host and the selection on the basis of phenotype.

The probability to increase the hit rate of the desired gene can be achieved by sample enrichment prior to library construction, development of new sensitive assays, automated high throughput screening. Lammle et al. (2007) enhanced the hit rate using dual orientation promoter. The choice of suitable screening methods plays a significant role in hitting the target gene.

Recently, a novel operon involved in desulfurization of dibenzothiophenes was identified by metagenomic screening by cloning the amplified gene in *E. coli* DH5α cells. The expressed gene product successfully desulfurizes dibenzothiophenes (Abbasian et al., 2016). Similarly, a novel lipase gene was isolated from a soil waste water treatment plant. The high hit rate was observed in the clones due to a quality of soil sample, which was fat contaminated soil and prokaryotic enriched DNA. A novel xylanase gene was isolated for the first time using a metagenomic approach that is alkaline and thermostable. The recombinant xylanase was applicable in paper and pulp industry, pulp bleaching and generating xylooligosaccharides from the abundantly available agro-residues (Verma et al., 2013). A cold active esterases were also found from a soil sample of Artic region, with their optima at 20°C and 30°C (Yu et al., 2011). Metagenomics has also established its importance in healthcare where Pehrsson et al., 2013 found rich diversity in antibiotic resistance genes with many previously unknown sequences. A study was conducted for the first time from mangrove soil to target bacterial laccase gene using metagenomic approach. The recombinant enzyme obtained was highly soluble and alkaline stable that makes it a good candidate for biobleaching industry (Ye et al., 2010).

There are various reports on success and contribution of functional metagenomics *viz*. discovery of various enzyme (**Table 2**) antimicrobials, bioremediation of recalcitrant compounds etc.

**Table 2:** Exploratory overview of various metagenomic genes and their cloning strategies.

S. No	Target gene	Source	Host	Cloning vector	Expression vector	Gene size	Reference
1	β-lactamases	Soil	*E. coli*	–	pCF430, pCC1BAC and pCC1FOS	42 kb	Allen et al., 2009
2	Cold adapted Xylanase	Antartic sea water	*E. coli*	–	pET22b		Marx et al., 2007
3	Lipase/ esterase	Bovine rumen	*E. coli*	–	pTrcHis TOPO vector	30–35 kb	Prive et al., 2015
4	Lipase	Waste water treatment plant	*E. coli*	–	pET28a	20–40 kb	Glogauer et al., 2002
5	Alkaline protease	Forest soil	*E. coli*	pHT01	pET-30	4–20 kb	Biver et al., 2013
6	Alkaline protease	Goat skin surface	*E. coli*	–	pUC19	3.8 kb	Paul et al., 2011
7	Pectinase	Soil of Hot spring Manikaran	*E. coli*	pGEMT	pQE30		Singh et al., 2012
8	Cellulase	German grassland soil	*E. coli*	pLC01	pET101/D	23–29 kb	Nacke et al., 2012
9	Amylase	Soil	*E. coli*	–	pUC 19	3–5 kb	Delavat et al., 2012
10	Phytase	Grass carp	*E. coli*	pGEMT	pET		Huang et al., 2009
11	Esterase	South China Sea	*E. coli*	pIndigoBAC- 5 vector	pUC19	70 kb	Chu et al., 2008
12	Lipase	Marine sponge	*E. coli*	pCC1FOS	pBAD/mycHis vector	40 kb	Selvin et al., 2012
13	Lipase	Sea sediment	*E. coli*	pCC1FOS	pGEX-6P-3 vector	35.4 kb	Hardeman and Sjoling, 2007
14	Protease	Antartica soil	*E. coli*	pCC1BAC	pCC1BAC		Berlemont et al., 2009
15	α amylase	Ikaite columns of SW Greenland	*E. coli*	mod.pGNS-BAC	pET21b	15 kb	Vester et al., 2014
16	β glucosidase	Ikaite columns of SW Greenland	*E. coli*	mod.pGNS-BAC	pET21b	15 kb	Rabausch et al., 2013

There has been large a contribution in the study of hydrolases but enzymes other than hydrolases from metagenomes are still in their infancy. Since only a very few reliable screening procedures are available that allow the rapid screening of large clone libraries. A new screening system was developed for the discovery of flavonoid-modifying enzymes based on high-performance thin-layer chromatography (HPTLC). This metagenome extract thin-layer chromatography analysis (META) allows the rapid detection of glycosyltransferase (GT) and also other flavonoid-modifying activities. This highly sensitive method can detect 4 ng of modified flavonoid molecules and have screened two novel UDP glycosyltransferase (UGT) genes from two different metagenomic preparations (Rabausch et al., 2013).

### Identification by Randomly Mass Sequencing

The term metagenomics blends two words: *meta-*analysis, a means of statistical analysis of the outcome of two distinct analyses, and *genomics*, i.e., analysis of genetic make-up (Rondon et al., 2000). The foremost objective of any metagenome sequencing project is to uncover and characterize particular community, chiefly “who’s there?”, “what are they doing?”, involving three main aspects: (1) composition/structure of the community, their genetic and phylogenetic relatedness, (2) role of each member within the community, and (3) intra-species or intra-population heterogeneity of the genes.

Initially, metagenomics confined only for diversity study but with time, it has progressed and found its application in various areas. The technique is being employed to uncover the functional properties of microorganism in a community, revealing enzyme with novel catalytic activity, antibiotic therapies, genes that are involved in bioremediation, dyestuff processing, lignocellulosic treatment, and biobleaching of paper pulp (Ye et al., 2010; Kumar et al., 2016). There are numerous studies on various unexplored samples, a study on Lonar soda lake sediment from which a significant percentage (11%) of unclassified species has been reported using bacterial tag-encoded FLX amplicon pyrosequencing (bTEFAP) (Dudhagara et al., 2015). Another study, which also detected similar percentage (10.69%) of unclassified prokaryotes from Tulsi Shyam hot spring, India (Ghelani et al., 2015). It is to be noted that such studies play a crucial role for the discovery of novel enzymes (**Table 2**).

Metagenomics has clustered environmental biology, functional biology, microbial physiology and sustainable development under one single frame. The genetic make-up of the community elucidates the type of flora, their functional role in the environment and the effectual metabolism of the species. The richness or deficiency of the particular species in the population in the particular area reveals the essence of a particular environment. Understanding the function, metabolism and succession of microorganisms helps to maintain the niche. Illeghems et al. (2015) analyzed metabolic capabilities of cocoa bean fermentation bacterial community and evaluated the network of cycles between the microorganisms. In an another study, seasonal change in microbial diversity was seen using metagenomics by Illumina Miseq platform and analyzed using MG-RAST, STAMP tool (Yadav et al., 2015). There has been an immense progress in the technology that renders such huge metagenomic data to be processed authentically. Singh and Shukla, 2015 summarize techniques and software to analyze the metagenomic data. Furthermore, studies on computational enzyme docking also elucidated the characterization of the efficient substrates for the enzymes (Karthik and Shukla, 2012; Singh and Shukla, 2012; Karumuri et al., 2015; Singh et al., 2016).

The analysis of sequences is performed by a sequence of steps with the foremost objective to filter the data. The filtered data can be analyzed by (1) Marker gene analysis, which involves comparing the metagenomic reads to a database consisting of gene families bearing specific genetic markers, the marker gene in the read can be spotted and reads are classified on the basis of homology to respective gene marker. Most commonly used marker genes are rRNA genes justifying ‘who is there?’ or protein coding sequences indicating ‘what are they doing?’ There are various types of software to taxonomically annotate the metagenomes *viz*. MetaPhlAn, AMPHORA, MetaPhyler, PhyloSift, PhylOTU. (2) Binning, a process of grouping reads to slot them to operational taxonomic units. Methods of binning are based either on compositional features or alignment or both. (a) compositional binning, which uses composition of sequences *viz* tags like rec A, rpo B, 16S rRNA to cluster the metagenomic reads into taxon. (b) similarity binning, relies on the alignment of the sequences against the known reference sequence. The aligned reads are clustered into the respective taxa. The softwares mainly used are MEGAN, MG-RAST, CARMA. (c) fragment recruitment, in which, reads are aligned to nearly identical genome sequences to produce metagenomic coverage estimates of the genome. There are several tools that help to map the reads *viz.* MOSAIK, Genometa, SOAP, BWA, CLC, RefCov. (3) Assembly reads that posses nearly identical sequences at their ends are linked to form contig or complete genome. Each successive sequencing project adds to the previous because of a collection of large data from the diverse environment. In a study conducted in acid mine drainage, a group of genomes was reconstructed belonging to unculturable using random shotgun sequencing of DNA from the biofilm (Siegert et al., 2003). Recently, a broad survey of the viral assemblages inhabiting the marine invertebrates revealed that different invertebrate groups harbor distinct viral assemblages (Gudenkauf and Hewson, 2016).

**FIGURE 4:**
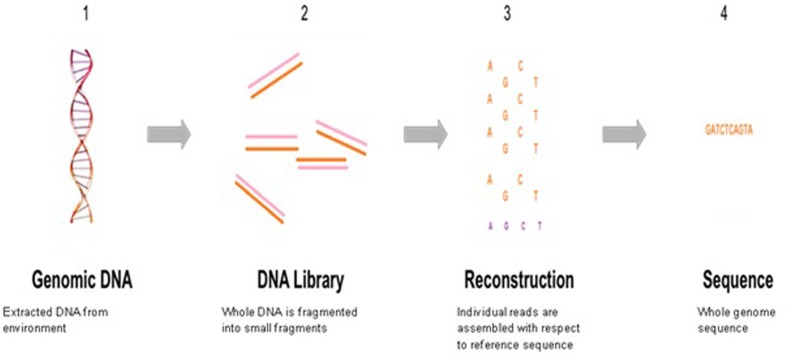
**A snapshot of traditional whole genome sequencing**.

The rapid expansion of new sequencing technologies and sequence searching tools has enabled large-scale functional exploration of numerous microbial ecosystems. Next generation sequencing has taken over traditional sequencing methods in terms of high-throughput, low costing, allowing deeper and clear prospective into microbial community diversity composition. NGS has the potential for complete profiling of microbial communities from extreme samples, uncover new species, and investigate the response of microbial populations under changing conditions. Environmental metagenomics as a field was extremely limited prior to the advent of next-generation sequencing (NGS). An overview of whole genome sequencing is shown in **Figure 4**.

### Improvement of Enzymatic Catalysis by Microbial Cell Surface Display

**FIGURE 5:**
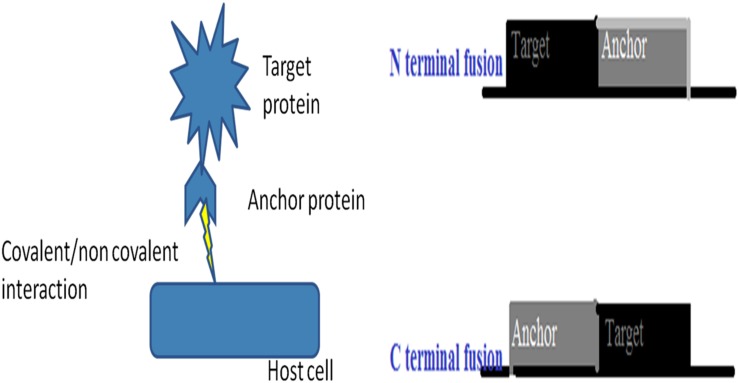
**A snapshot of Microbial cell surface display**.

**FIGURE 6:**
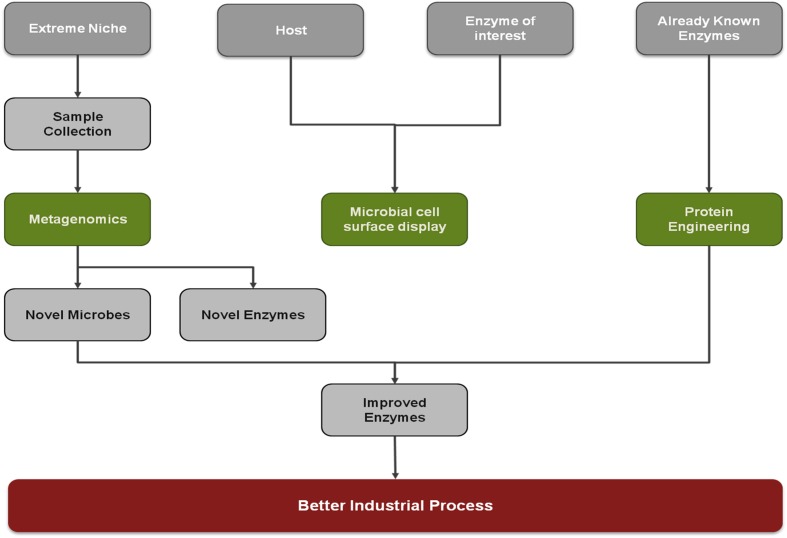
**A snapshot of techniques to improve the industrial process**.

Microbial cell surface display is a technique developed to empower any industrial or biotechnological process. Cell-surface display provides an opportunity to display peptides and proteins of interest on the surface of microbial cells by fusing them with the anchoring motifs (**Figure 5**). It is a simultaneous expression of two or more proteins in a single cell system that could have the cumulative effect on the process (Tanaka and Kondo, 2015). The protein of interest (target protein or fusion protein) is fused to anchor protein via tethering on the cell wall and expressed in a host cell. The orientation of the target protein with respect to anchor protein is important for its activity. For example, in N-terminal fusion, the N-terminus of an anchor protein is fused to the C-terminus of a target protein (**Figure 2**). Conversely, for C-terminal fusion, the C-terminus of an anchor protein is fused to the N-terminus of a target protein. The technique has a wide range of biotechnological and industrial applications, including: live vaccine development, a recombinant vaccine against parapoxvirus, orf virus (ORFV) was developed that causes superficial skin lesions in infected humans and grazing animals. The *Echinococcus granulosus* antigen EG95 was genetically fused on the surface proteins of a host cell and recombinants were prepared. The recombinants successfully reduced the infectivity during *in vitro* assay and good antibody response was observed in the inoculated sheep (Tan et al., 2012). In a similar study, the live oral vaccine against chicken coccidiosis was developed for the first time using yeast *Saccharomyces cerevisiae* as a host strain. *Eimeria tenella* EtMic2 protein acted as a fusion protein and provided a humoral as well as cell mediated immunological response (Sun et al., 2014). A snapshot of techniques involved in the improvement of industrial process is depicted in **Figure 6**. In a recent study, a group of researchers developed a nano shuttles by engineering the exosomes and exploited their application in targeted drug delivery, as well as exosome-mediated vaccine and therapy utilizing cell surface display technology (Stickney et al., 2016). Another technique based on cell surface display was developed to inhibit HIV infection by using an antibody as fusion peptide together with autotransporter β-barrel domain of IgAP gene from *Neisseria gonorrhoeae*. The engineered bacteria successfully captured HIV-1 particles via surface-binding and inhibit HIV-1 infection in cell culture (Wang et al., 2015).

The lipase was expressed on the surface of yeast cell and it was found that engineered yeast showed high-performance characteristics and variant utilizations. It depicts its application in various areas like a synthesis of esters, PUFA enrichment, resolution of chiral drugs, organic synthesis and biofuels (Liu et al., 2014a). Another lipase from *Staphylococcus haemolyticus* L62, displayed on the cell surface of *E. coli* using an autotransporter protein of *Pseudomonas putida* EstAβ8 as an anchoring motif showed its application in biodiesel production and yield of nearly 89.4% after a 96 h reaction at 30°C (Kim et al., 2013). Similarly, nitrilase from *Thermotoga maritima* MSB8 was also surface displayed on *Bacillus* spores. The study concludes that surface display of enzymes on the spore of *B. subtilis* might be an effective method for enzyme immobilization and help to meet the ever-increasing industrial demand for preparation and stabilization of biocatalysts (Chen et al., 2016). A bioremediation approach was established for the first time using triphenylmethane reductase (TMR). The enzyme was surface displayed on *E. coli* using ice nucleation protein as an anchor. It was reported that the decolorization rate for the malachite green of this engineered strain is the highest so far, with 640 μmol min^-1^ g^-1^ dry weight cells (Gao et al., 2014). A biocatalyst for biosensor has been developed for assay of glutamate by fusing thermophilic glutamate dehydrogenase Gldh on *E. coli*. The glutamate assay plays an important role in the diagnosis of many neurological disorders like epilepsy, amyotrophic lateral sclerosis or Parkinson’s disease. The enzyme was quite stable and ion tolerant (Song et al., 2015).

## Conclusion

A wide range of microorganisms from extreme niche is known for producing novel catalyst, extremozymes. They are essentially important to meet industrial demand and switch from chemical based products to biological products. Metagenomics have been initiated to uncover such novel enzyme producers. A few successful attempts have been made to engineer the natural enzyme but progress still has to be made to develop the robust approaches. The microbial surface display is still another approach to make the process more efficient and accessible. The enzyme technology is still in the progressive phase and expected to achieve spectacular outcomes in nearest future. System biology is also proving as one of the important technique decipher microbial interactions (Baweja et al., 2015).

## Author Contributions

All authors listed, have made substantial, direct and intellectual contribution to the work, and approved it for publication.

## Conflict of Interest Statement

The authors declare that the research was conducted in the absence of any commercial or financial relationships that could be construed as a potential conflict of interest.

## References

[B1] Abbasian F. Lockington R. Megharaj M. Naidu R. (2016). Identification of a new operon involved in desulfurization of dibenzothiophenes using a metagenomic study and cloning and functional analysis of the genes. *Enzyme Microb. Technol.* 87 24–28. 10.1016/j.enzmictec.2016.02.009 27178791

[B2] Allen H. K. Moe L. A. Rodbumrer J. Gaarder A. Handelsman J. (2009). Functional metagenomics reveals diverse beta-lactamases in a remote Alaskan soil. *ISME J.* 3 243–251. 10.1038/ismej.2008.86 18843302

[B3] Amico S. D. Collins T. Gerday C. (2006). Psychrophilic microorganisms: challenges for life. *EMBO Rep.* 7 385–389. 10.1038/sj.embor.7400662 16585939 PMC1456908

[B4] Baweja M. Singh P. K. Shukla P. (2015). “Enzyme technology, functional proteomics and systems biology towards unraveling molecular basis for functionality and interactions in biotechnological processes,” in *Frontier Discoveries and Innovations in Interdisciplinary Microbiology* ed. Shukla P. (Berlin: Springer-Verlag), 207–212.

[B5] Berlemont R. Pipers D. Delsaute M. Angiono F. Feller G. Galleni M. (2009). Exploring the Antarctic soil metagenome as a source of novel cold-adapted enzymes and genetic mobile elements. *Rev. Argent. Microbiol.* 43 94–103. 10.1590/S0325-75412011000200005 21731970

[B6] Biver S. Portetelle D. Vandenbol M. (2013). Characterization of a new oxidant-stable serine protease isolated by functional metagenomics. *Springerplus* 2:410 10.1186/2193-1801-2-410 PMC3765597 24024096

[B7] Cesarini S. Bofill C. Javier Pastor F. I. Reetz M. T. Diaz P. (2012). A thermostable variant of *P. aeruginosa* cold-adapted LipC obtained by rational design and saturation mutagenesis. *Process Biochem.* 47 2064–2071. 10.1016/j.procbio.2012.07.023

[B8] Chen H. Chen Z. Ni Z. Tian R. Zhang T. Jia J. (2016). Display of *Thermotoga maritima* MSB8 nitrilase on the spore surface of *Bacillus subtilis* using out coat protein CotG as the fusion partner. *J. Mol. Catal. B Enzym.* 123 73–80. 10.1016/j.molcatb.2015.11.002

[B9] Chi M. C. Chen Y. H. Wu T. J. Lo H. F. Lin L. L. (2010). Engineering of a truncated α-amylase of *Bacillus* sp. strain TS-23 for the simultaneous improvement of thermal and oxidative stabilities. *J. Biosci. Bioeng.* 109 531–538. 10.1016/j.jbiosc.2009.11.012 20471589

[B10] Chu X. He H. Guo C. Sun B. (2008). Identification of two novel esterases from a marine metagenomic library derived from South China Sea. *Appl. Microbiol. Biotechnol.* 80 615–625. 10.1007/s00253-008-1566-3 18600322

[B11] Collonnier C. Nogue F. Casacuberta J. M. (2015). “Targeted genetic modification in crops using site-directed nucleases,” in *Genetically Modified Organisms in Food* R. R. Watson and V. Preedy (Cambridge: Academic Press), 133–145.

[B12] Delavat F. Phalip V. Forster A. Plewniak F. Lett M. C. Lievremont D. (2012). Amylases without known homologues discovered in an acid mine drainage: significance and impact. *Sci. Rep.* 2:354 10.1038/srep00354 PMC3319935 22482035

[B13] Dudhagara P. Ghelani A. Patel R. Chaudhari R. Bhatt S. (2015). Bacterial tag encoded FLX titanium amplicon pyrosequencing (bTEFAP) based assessment of prokaryotic diversity in metagenome of Lonar soda lake, India. *Genom. Data* 4 8–11. 10.1016/j.gdata.2015.01.010 26484168 PMC4535755

[B14] Eijsink V. G. H. Veltman O. R. Aukema W. Vriend G. Venema G. (1995). Structural determinants of the stability of thermolysin-like proteinases. *Nat. Struct. Biol.* 2 374–379. 10.1038/nsb0595-374 7664094

[B15] Fang Z. M. Li T. L. Chang F. Zhou P. Fang W. Hong Y. Z. (2012). A new marine bacterial laccase with chloride-enhancing, alkaline-dependent activity and dye decolorization ability. *Bioresour. Technol.* 111 36–41. 10.1016/j.biortech.2012.01.172 22377476

[B16] Feller G. (2013). Psychrophilic enzymes: from folding to function and biotechnology. *Scientifica* 2013:512840 10.1155/2013/512840 PMC3820357 24278781

[B17] Gao F. Ding H. Feng Z. Liu D. Zhao Y. (2014). Functional display of triphenylmethane reductase for dye removal on the surface of *Escherichia coli* using N-terminal domain of ice nucleation protein. *Bioresour. Technol.* 169 181–187. 10.1016/j.biortech.2014.06.093 25058292

[B18] Ghelani A. Patel R. Mangrol A. Dudhagara P. (2015). Cultivation-independent comprehensive survey of bacterial diversity in Tulsi Shyam Hot Springs, India. *Genom. Data* 4 54–56. 10.1016/j.gdata.2015.03.003 26484176 PMC4536058

[B19] Glogauer A. Martini V. P. Faoro H. Couto G. H. Santos M. M. Monteiro R. A. (2002). Identification and characterization of a new true lipase isolated through metagenomic approach. *Microbial Cell Fact.* 10:54 10.1186/1475-2859-10-54 PMC3161859 21762508

[B20] Gudenkauf B. M. Hewson I. (2016). Comparative metagenomics of viral assemblages inhabiting four phyla of marine invertebrates. *Front. Mar. Sci.* 3:206 10.3389/fmars.2016.00023

[B23] Gupta S. K. Shukla P. (2015). Advanced technologies for improved expression of recombinant proteins in bacteria: perspectives and applications. *Crit. Rev. Biotechnol.* 18 1–10. 10.3109/07388551.2015.1084264 26384140

[B24] Hardeman F. Sjoling S. (2007). Metagenomic approach for the isolation of a novel low-temperature-active lipase from uncultured bacteria of marine sediment. *FEMS Microbiol. Ecol.* 59 524–534. 10.1111/j.1574-6941.2006.00206.x 17328767

[B25] Huang H. Shi P. Wang Y. Luo H. Shao N. Wang G. (2009). Diversity of beta-propeller phytase genes in the intestinal contents of grass carp provides insight into the release of major phosphorus from phytate in nature. *Appl. Environ. Microbiol.* 75 1508–1516. 10.1128/AEM.02188-08 19151187 PMC2655465

[B26] Illeghems K. Weckx S. Vuyst L. D. (2015). Applying meta-pathway analyses through metagenomics to identify the functional properties of the major bacterial communities of a single spontaneous cocoa bean fermentation process sample. *Int. J. Food Microbiol.* 50 54–63. 10.1016/j.fm.2015.03.005 25998815

[B27] Jackson S. A. Borchert E. Gara F. Dobson A. D. W. (2015). Metagenomics for the discovery of novel biosurfactants of environmental interest from marine ecosystems. *Curr. Opin. Biotechnol.* 33 176–182. 10.1016/j.copbio.2015.03.004 25812477

[B28] Jacquiod S. Demaneche S. Franqueville L. Ausec L. Xu Z. Delmont T. O. (2014). Characterization of new bacterial catabolic genes and mobile genetic elements by high throughput genetic screening of a soil metagenomic library. *J. Biotechnol.* 190 18–29. 10.1016/j.jbiotec.2014.03.036 24721211

[B29] Jaouadi B. Aghajari N. Haser R. Bejar S. (2010). Enhancement of the thermostability and the catalytic efficiency of *Bacillus pumilus* CBS protease by site-directed mutagenesis. *Biochimie* 92 360–369. 10.1016/j.biochi.2010.01.008 20096326

[B30] Jon E. N. Welch M. Giver L. Bueno M. Cherry J. R. Borchert T. V. (1999). DNA shuffling of subgenomic sequences of subtilisin. *Nat. Biotechnol.* 17 893–896. 10.1038/12884 10471932

[B31] Kahlke T. Thorvaldsen S. (2012). Molecular characterization of cold adaptation of membrane proteins in the vibrionaceae core-genome. *PLoS ONE* 7:e51761 10.1371/journal.pone.0051761 PMC3524096 23284762

[B32] Kano H. Taguchi S. Momose H. (1997). Cold adaptation of a mesophilic serine protease, subtilisin, by in vitro random mutagenesis. *Appl. Microbiol. Biotechnol.* 47 46–51. 10.1007/s002530050886 9035410

[B33] Karthik M. V. K. Shukla P. (2012). *Computational Strategies Towards Improved Protein Function Prophecy of Xylanases from Themomyces Lanuginosus.* Berlin: Springer 10.1007/978-1-4614-4723-8

[B34] Karumuri S. Singh P. K. Shukla P. (2015). In silico analog design for terbinafine against *Trichophyton rubrum*: a preliminary study. *Indian J. Microbiol.* 55 333–340. 10.1007/s12088-015-0524-x 26063944 PMC4456508

[B35] Kim S. Song J. K. Kim H. K. (2013). Cell surface display of *Staphylococcus haemolyticus* L62 lipase in *Escherichia coli* and its application as a whole cell biocatalyst for biodiesel production. *J. Mol. Catal. B Enzym.* 97 54–61. 10.1016/j.molcatb.2013.07.017

[B36] Kuddus M. Ramteke W. P. (2011). Production optimization of an extracellular cold-active alkaline protease from *Stenotrophomonas maltophilia* MTCC 7528 and its application in detergent industry. *Afr. J. Microbiol. Res.* 5 809–816.

[B37] Kulakova L. Galkin A. Nakayama T. Nishino T. Esaki N. (2003). Improvement of thermostability of cold-active serine alkaline protease from the psychrotrophic bacterium *Shewanella* sp. strain Ac10 by rational mutagenesis. *J. Mol. Catal. B Enzym.* 22 113–117. 10.1016/S1381-1177(03)00012-2

[B38] Kumar V. Marín-Navarro J. Shukla P. (2016). Thermostable microbial xylanases for pulp and paper industries: trends, applications and further perspectives. *World J. Microbiol. Biotechnol.* 32 1–10. 10.1007/s11274-015-2005-0 26754672

[B39] Lammle K. Zipper H. Breuer M. Hauer B. Buta C. Brunner H. (2007). Identification of novel enzymes with different hydrolytic activities by metagenome expression cloning. *J. Biotechnol.* 127 575–592. 10.1016/j.jbiotec.2006.07.036 16963141

[B40] Li J. J. Pei X. Q. Zhang S. B. Wu Z. L. (2015). Improving the thermostability of feruloyl esterase by DNA shuffling and site-directed mutagenesis. *Process Biochem.* 50 1783–1787. 10.1016/j.procbio.2015.08.009

[B41] Li Z. Chen X. Guo S. Zhang H. Dong H. Wu G. (2015). Engineering of Harobin for enhanced fibrinolytic activity obtained by random and site-directed mutagenesis. *Protein Expr. Purif.* 10.1016/j.pep.2015.09.010 [Epub ahead of print]. 26363113

[B42] Lidan Y. Hua Z. Zhi L. Wu J. C. (2013). Improved acid tolerance of *Lactobacillus pentosus* by error-prone whole genome amplification. *Bioresour. Technol.* 135 459–463. 10.1016/j.biortech.2012.10.042 23182040

[B43] Lillford P. J. Holt C. B. (2002). In vitro uses of biological cryoprotectants. *Philos. Trans. R. Soc. Lond. B Biol. Sci.* 357 945–951. 10.1098/rstb.2002.1083 12171658 PMC1692994

[B44] Liu Y. Zhang R. Lian Z. Wang S. Wright A. T. (2014a). Yeast cell surface display for lipase whole cell catalyst and its applications. *J. Mol. Catal. B Enzym.* 106 17–25. 10.1016/j.molcatb.2014.04.011

[B45] Liu Y. Zhang T. Zhang Z. Sun T. Wang J. Lu F. (2014b). Improvement of cold adaptation of Bacillus alcalophilusalkaline protease by directed evolution. *J. Mol. Catal. B Enzym.* 106 117–125. 10.1016/j.molcatb.2014.05.005

[B46] Mabrouk S. B. Ayadi D. Z. Hlima H. B. Bejar S. (2013). Thermostability improvement of maltogenic amylase MAUS149 by error prone PCR. *J. Biotechnol.* 168 601–606. 10.1016/j.jbiotec.2013.08.026 23994264

[B47] Marx J. C. Collins T. Amico S. D. Feller G. (2007). Cold-adapted enzymes from marine antarctic microorganisms. *Mar. Biotechnol.* 9 293–304. 10.1007/s10126-006-6103-8 17195087

[B48] Melzer S. Sonnendecker C. Follner C. Zimmermann W. (2015). Stepwise error-prone PCR and DNA shuffling changed the pH activity range and product specificity of the cyclodextrin glucanotransferase from an alkaliphilic *Bacillus* sp. *FEBS Open Bio* 5 528–534. 10.1016/j.fob.2015.06.002 PMC4491590 26155461

[B49] Minamoto T. Wada E. Shimizu I. (2012). A new method for random mutagenesis by error-prone polymerase chain reaction using heavy water. *J. Biotechnol.* 157 71–74. 10.1016/j.jbiotec.2011.09.012 21963598

[B50] Nacke H. Engelhaupt M. Brady S. Fischer C. Tautzt J. Daniel R. (2012). Identification and characterization of novel cellulolytic and hemicellulolytic genes and enzymes derived from German grassland soil metagenomes. *Biotechnol. Lett.* 34 663–675. 10.1007/s10529-011-0830-2 22187078 PMC3298741

[B51] Nurdiani D. Ito M. Maruyama T. Terahara T. Mori T. Ugawa S. (2015). Analysis of bacterial xylose isomerase gene diversity using gene-targeted metagenomics. *J. Biosci. Bioeng.* 120 174–180. 10.1016/j.jbiosc.2014.12.022 25656071

[B52] Paul L. P. Thangamani R. Paramasamy G. (2011). Identification and characterization of alkaline serine protease from goat skin surface metagenome. *AMB Express.* 1:3 10.1186/2191-0855-1-3 PMC3159910 21906326

[B53] Pehrsson E. Forsberg K. J. Gibson M. K. Ahmadi S. Dantas G. (2013). Novel resistance function uncovered using functional metagenomic investigations of resistance reservoirs. *Front. Microbiol.* 4:145 10.3389/fmicb.2013.00145 PMC3675766 23760651

[B54] Prive F. Newbold C. J. Kaderbhai N. N. Girdwood S. G. Golyshina O. V. Golyshin P. N. (2015). Isolation and characterization of novel lipases/esterases from a bovine rumen metagenome. *Appl. Microbiol. Biotechnol.* 99 5475–5485. 10.1007/s00253-014-6355-6 25575887 PMC4464377

[B55] Rabausch U. Juergensen J. Ilmberger J. Bohnke S. Fischer S. Schubach B. (2013). Functional screening of metagenome and genome libraries for detection of novel flavonoid-modifying enzymes. *Appl. Environ. Microbiol.* 79 4551–4563. 10.1128/AEM.01077-13 23686272 PMC3719526

[B56] Ramli A. M. Muhammad N. M. Rabu A. Munir A. Murad A. Diba F. A. B. (2011). Molecular cloning, expression, and biochemical characterisation of a cold-adapted novel recombinant chitinase from Glaciozyma antarctica PI12. *Microb. Cell Fact.* 10:94 10.1186/1475-2859-10-94 PMC3226447 22050784

[B57] Rasila T. S. Pajunen M. I. Savilahti H. (2009). Critical evaluation of random mutagenesis by error-prone polymerase chain reaction protocols, *Escherichia coli* mutator strain, and hydroxylamine treatment. *Anal. Biochem.* 388 71–80. 10.1016/j.ab.2009.02.008 19454214

[B58] Rondon M. R. August P. R. Bettermann A. D. Brady S. F. Grossman T. H. Liles M. R. (2000). Cloning the soil metagenome: a strategy for accessing the genetic and functional diversity of uncultured microorganisms. *Appl. Environ. Microbiol.* 66 2541–2547. 10.1128/AEM.66.6.2541-2547.2000 10831436 PMC110579

[B59] Sabree Z. N. Rondon M. R. Handelsman J. (2009). *Metagenomics. Encyclopedia of Microbiology 3rd Edn.* Waltham, MA: Academic Press 622–632.

[B60] Selvin J. Kennedy J. Lejon D. P. H. Kiran G. S. Dobson A. D. W. (2012). Isolation identification and biochemical characterization of a novel halo-tolerant lipase from the metagenome of the marine sponge *Haliclona* simulans. *Microb. Cell Fact.* 11:72 10.1186/1475-2859-11-72 PMC3544137 22657530

[B61] Sen S. Dasu V. V. Mandal B. (2007). Developments in directed evolution for improving enzyme functions. *Appl. Biochem. Biotechnol.* 143 212–223. 10.1007/s12010-007-8003-4 18057449

[B62] Shukla P. Garai D. Zafar M. Gupta K. Shrivastava S. (2007). Process parameters optimization for lipase production by *Rhizopus oryzae* kg-10 under submerged fermentation using response surface methodology. *J. Appl. Sci. Environ. Sanit.* 2 93–103.

[B63] Shukla P. Gupta K. (2007). Ecological screening for lipolytic molds and process optimization for lipase production from *Rhizopus oryzae* KG-5. *J. Appl. Sci. Environ. Sanit.* 2 35–42.

[B64] Siegert M. J. Tranter M. Evans J. C. E. Priscu J. C. Lyons W. B. (2003). The hydrochemistry of Lake Vostok and the potential for life in Antarctic subglacial lakes. *Hydrol. Process.* 17 795–814. 10.1002/hyp.1166

[B65] Singh P. Shukla P. (2015). Systems biology as an approach for deciphering microbial interactions. *Brief Funct. Genomics.* 14 166–168. 10.1093/bfgp/elu023 24994863

[B66] Singh P. K. Joseph J. Goyal S. Grover A. Shukla P. (2016). Functional analysis of the binding model of microbial inulinases using docking and molecular dynamics simulation. *J. Mol. Model.* 22 1–7. 10.1007/s00894-016-2935-y 26956120

[B67] Singh P. K. Shukla P. (2012). Molecular modeling and docking of microbial inulinases towards perceptive enzyme-substrate interactions. *Indian J. Microbiol.* 52 373–380. 23997327 10.1007/s12088-012-0248-0 PMC3460115

[B68] Singh R. Dhawan S. Singh K. Kaur J. (2012). Cloning, expression and characterization of a metagenome derived thermoactive/thermostable pectinase. *Mol. Biol. Rep.* 39 8353–8361. 10.1007/s11033-012-1685-x 22711301

[B69] Song J. Liang B. Han D. Tang X. Lang Q. Fenga R. (2015). Bacterial cell-surface displaying of thermo-tolerant glutamate dehydrogenase and its application in l-glutamate assay. *Enzyme Microb. Tech.* 70 72–78. 10.1016/j.enzmictec.2014.12.002 25659635

[B70] Stickney Z. Losacco J. McDevitt S. Zhang Z. Lu B. (2016). Development of exosome surface display technology in living human cells. *Biochem. Biophys. Res. Commun.* 472 53–59. 10.1016/j.bbrc.2016.02.058 26902116

[B71] Struvay C. Feller G. (2012). Optimization to low temperature activity in psychrophilic enzymes. *Int. J. Mol. Sci.* 13 11643–11665. 10.3390/ijms130911643 23109875 PMC3472767

[B72] Sun H. Wang L. Wang T. Zhang J. Liu Q. Chen P. (2014). Display of *Eimeria tenella* EtMic2 protein on the surface of *Saccharomyces cerevisiae* as a potential oral vaccine against chicken coccidiosis. *Vaccine* 32 1869–1876. 10.1016/j.vaccine.2014.01.068 24530147

[B73] Tan J. L. Ueda N. Heath D. Mercer A. A. Fleminga S. B. (2012). Development of orf virus as a bifunctional recombinant vaccine: surface display of *Echinococcus granulosus* antigen EG95 by fusion to membrane structural proteins. *Vaccine* 30 398–406. 10.1016/j.vaccine.2011.10.079 22085551

[B74] Tanaka T. Kondo A. (2015). Cell surface engineering of industrial microorganisms for biorefining applications. *Biotechnol. Adv.* 33 1403–1411. 10.1016/j.biotechadv.2015.06.002 26070720

[B75] Tiwari R. Singh S. Shukla P. Nain L. (2014). Novel cold temperature active β-glucosidase from *Pseudomonas lutea* BG8 suitable for simultaneous saccharification and fermentation. *RSC Adv.* 4 58108–58115. 10.1039/C4RA09784J

[B76] Venter J. C. Remington K. Heidelberg J. F. Halpern A. L. Rusch D. Eisen J. A. (2004). Environmental genome shotgun sequencing of the Sargasso sea. *Science* 304 66–74. 10.1126/science.1093857 15001713

[B77] Verma D. Kawarabayasi Y. Miyazaki K. Satyanarayana T. (2013). Cloning, expression and characteristics of a novel alkalistable and thermostable xylanase encoding gene (Mxyl) retrieved from compost-soil metagenome. *PLoS ONE* 8:e52459 10.1371/journal.pone.0052459 PMC3561394 23382818

[B78] Vester J. K. Glaring M. A. Stougaard P. (2014). Discovery of novel enzymes with industrial potential from a cold and alkaline environment by a combination of functional metagenomics and culturing. *Microb. Cell Fact.* 13:72 10.1186/1475-2859-13-72 PMC4035831 24886068

[B79] Wang H. K. Sun Y. Chen C. Sun Z. Zhou Z. C. Shen F. (2013). Genome shuffling of *Lactobacillus plantarum* for improving antifungal activity. *Food Control* 32 341–347. 10.1016/j.foodcont.2012.12.020

[B80] Wang H. Y. Liu D. M. Liu Y. Cheng C. F. Ma Q. Y. Huang Q. (2007). Screening and mutagenesis of a novel *Bacillus pumilus* strain producing alkaline protease for dehairing. *Lett. Appl. Microbiol.* 44 1–6. 10.1111/j.1472-765X.2006.02039.x 17209806

[B81] Wang L. X. Mellon M. Bowder D. Quinn M. Shea D. Wood C. (2015). *Escherichia coli* surface display of single-chain antibody VRC01 against HIV-1 infection. *Virology* 475 179–186. 10.1016/j.virol.2014.11.018 25482819 PMC4337897

[B82] Weng M. Deng X. Bao W. Zhu L. Wu J. Cai Y. (2015). Improving the activity of the subtilisin nattokinase by site-directed mutagenesis and molecular dynamics simulation. *Biochem. Biophys. Res. Commun.* 465 580–586. 10.1016/j.bbrc.2015.08.063 26291268

[B83] Wu G. Wang G. Ji J. Li Y. Gao H. Wu J. (2015). A chimeric vacuolar Na^+^/H^+^ antiporter gene evolved by DNA family shuffling confers increased salt tolerance in yeast. *J. Biotechnol.* 203 1–8. 10.1016/j.jbiotec.2015.02.033 25784157

[B84] Xie X. Huo W. Xia J. Xu Q. Chen N. (2012). Structure–activity relationship of a cold-adapted purine nucleoside phosphorylase by site-directed mutagenesis. *Enzyme Microb. Technol.* 51 59–65. 10.1016/j.enzmictec.2012.04.002 22579392

[B85] Xu H. Qin Y. Huang Z. Liu Z. (2014). Characterization and site-directed mutagenesis of an α-galactosidase from the deep-sea bacterium *Bacillus megaterium*. *Enzyme Microb. Technol.* 56 46–52. 10.1016/j.enzmictec.2014.01.004 24564902

[B86] Yadav T. C. Pal R. R. Shastri S. Jadeja N. B. Kapley A. (2015). Comparative metagenomics demonstrating different degradative capacity of activated biomass treating hydrocarbon contaminated wastewater. *Bioresour. Technol.* 188 24–32. 10.1016/j.biortech.2015.01.141 25727998

[B87] Yasukawa K. Inouye K. (2007). Improving the activity and stability of thermolysin by site-directed mutagenesis. *Biochim. Biophys. Acta* 1774 1281–1288. 10.1016/j.bbapap.2007.08.002 17869197

[B88] Ye J. Wen F. Xu Y. Zhao N. Long L. Sun H. (2015). Error-prone pcr-based mutagenesis strategy for rapidly generating high-yield influenza vaccine candidates. *Virology* 482 234–243. 10.1016/j.virol.2015.03.051 25899178 PMC4461506

[B89] Ye M. Li G. Liang W. Q. Liu Y. H. (2010). Molecular cloning and characterization of a novel metagenome-derived multicopper oxidase with alkaline laccase activity and highly soluble expression. *Appl. Microbiol. Biotechnol.* 87 1023–1031. 10.1007/s00253-010-2507-5 20358193

[B90] Yokoigawa K. Okubo Y. Soda K. Misono H. (2003). Improvement in thermostability and psychrophilicity of psychrophilic alanine racemase by site-directed mutagenesis. *J. Mol. Catal. B Enzym.* 23 389–395. 10.1016/S1381-1177(03)00103-6

[B91] Yu E. Y. Kwon M. A. Lee M. Oh J. Y. Choi J. E. Lee J. Y. (2011). Isolation and characterization of cold-active family VIII esterases from an arctic soil metagenome. *Appl. Microbiol. Biotechnol.* 90 573–581. 10.1007/s00253-011-3132-7 21318360

[B92] Zhang N. Suen W. C. Windsor W. Xiao L. Madison V. Zaks A. (2003). Improving tolerance of *Candida antarctica* lipase B towards irreversible thermal inactivation through directed evolution. *Protein Eng.* 16 599–605. 10.1093/protein/gzg074 12968077

[B93] Zhang Z. Akutsu J. Tsujimura M. Kawarabayasi Y. (2007). Increasing in archaeal GlcNAc-1-P uridyltransferase activity by targeted mutagenesis while retaining its extreme thermostability. *J. Biochem.* 141 553–562. 10.1093/jb/mvm058 17307792

[B94] Zhang Z. Shimizu Y. Kawarabayasi Y. (2015). Characterization of the amino acid residues mediating the unique amino-sugar-1-phosphate acetyltransferase activity of the archaeal ST0452 protein. *Extremophiles* 19 417–427. 10.1007/s00792-014-0727-9 25567746

